# Home-based transcutaneous electrical acupuncture-point stimulation for depressive symptoms in inflammatory bowel disease: a randomized feasibility study

**DOI:** 10.1097/MEG.0000000000003034

**Published:** 2025-10-29

**Authors:** Chongwen Huang, Wladyslawa Czuber-Dochan, Christine Norton

**Affiliations:** a Institute of Psychiatry, Psychology & Neuroscience; b Division of Care for Long Term Conditions, Reader in Nursing and Applied Health Research, Florence Nightingale Faculty of Nursing, Midwifery & Palliative Care; c Division of Care for Long Term Conditions, Florence Nightingale Faculty of Nursing, Midwifery and Palliative Care, London, UK

**Keywords:** acupuncture points, depression, digital health, fatigue, inflammatory bowel disease, mental health, mobile applications, pain, Patient Health Questionnaire, quality of life

## Abstract

**Background:**

Depressive symptoms are common in individuals with inflammatory bowel disease (IBD) and are associated with poor disease outcomes. Transcutaneous electrical acupuncture point stimulation (TEAS) is a noninvasive intervention with potential benefits for inflammation but remains untested in IBD. We developed a smartphone application for home-based TEAS self-administration and evaluated its feasibility, acceptability, and preliminary effects.

**Methods:**

In a randomized feasibility study, IBD patients with Patient Health Questionnaire-9 (PHQ-9) scores ≥ 8 were recruited through Crohn’s and Colitis UK. Participants were remotely trained to use the TEAS device, locate acupoints, and self-administer the treatment via the app. Group A completed 30-min daily sessions for 21 days from week 1, while Group B started in week 6. Outcomes [recruitment, retention, acceptability, depression, fatigue, pain, and quality of life (QoL)] were assessed at baseline, week 4, and week 9.

**Results:**

Of the 109 individuals of interest, 57 were assessed, 37 were eligible, and 36 were randomized. In Group A, 83% (15/18) completed ≥ 18 sessions, compared to 50% (9/18) in Group B. The questionnaire completion rates were 92% (*n* = 33) at baseline, 83% (*n* = 30) at 4 weeks, and 67% (*n* = 24) at 9 weeks. Most participants (81%) recommended TEAS. Preliminary analysis showed reduced depressive symptoms and improved QoL postintervention and at the follow-up. Postintervention, the mean PHQ-9 score decreased from 13.9 to 7.7 in Group A and from 14.2 to 6.5 in Group B.

**Conclusions:**

Home-based TEAS is feasible, acceptable, and has a clinical potential. A full-scale randomized controlled trial is needed to confirm its efficacy in the treatment of IBD-related depression.

## Introduction

The global incidence of inflammatory bowel disease (IBD) is increasing, particularly in children [1]. This growing burden is compounded by the high prevalence of comorbid depression, which affects approximately one-quarter of the patients [2]. Depression not only undermines patients’ well-being but is also associated with worse clinical outcomes, including more frequent IBD exacerbations, higher relapse rates, increased need for surgery, and greater healthcare resource utilization [3]. Adding to this challenge is the burden of living with IBD as many people are diagnosed at an early age, which affects their personal, educational, and professional development. Despite the importance of addressing depression as part of IBD management, adherence to antidepressant therapy is poor, and many patients discontinue treatment prematurely [4]. Complementary therapies, including acupuncture, have been utilized by over half of patients with IBD [5], reflecting an unmet therapeutic need within this population.

The high prevalence of mental health disorders among patients with IBD points to a complex interplay between the brain and the gut, extending beyond psychological and social factors [6]. Neuroinflammation-induced depression in IBD may result from peripheral inflammation, where inflammatory mediators from the inflamed gut cross the blood–brain barrier and activate microglial cells in the central nervous system either directly or indirectly [7]. Targeting these peripheral inflammatory mediators may help to alleviate depressive symptoms and offer a potential therapeutic pathway.

While acupuncture has demonstrated therapeutic benefits for various physical and mental health conditions, such as depression [8] and Crohn’s disease (CD) [8,9], traditional needle-based methods have certain limitations. Transcutaneous electrical acupoint stimulation (TEAS), a noninvasive technique rooted in traditional Chinese medicine supported by modern neuromodulation theory, offers a promising alternative. TEAS delivers mild electrical stimulation to acupuncture points and provides several advantages: it is safe, portable, capable of delivering consistent and quantifiable stimulation, and suitable for self-administration at home [10–12].

Commonly used acupoints are located on peripheral nerve trunks or branches, with varying selectivity and specificity [13]. Clinical trials have demonstrated the efficacy and safety of TEAS for mental health disorders [14–16] and gastrointestinal conditions [12,17,18], suggesting its potential as a therapeutic intervention for patients with IBD and depression. Despite its promise, TEAS has not been explored in IBD patients with depression, warranting further research to evaluate its feasibility and effectiveness.

In clinical trials of complementary medicine, understanding patient experiences is crucial for translating findings into practice [19]. To inform our study design, we conducted patient and public involvement consultations with three focus groups comprising 24 patients with IBD who self-reported depression. Their feedback led to an expansion of outcome measures to include pain and fatigue alongside depression and quality of life (QoL). We developed a mobile app and content management system to enable IBD patients with depressive symptoms to self-administer TEAS interventions while monitoring adherence. This feasibility study evaluated the acceptability and practicality of the research design of this digital TEAS intervention and made preliminary estimates of its clinical effectiveness.

## Materials and methods

### Study design and participants

This study was an open-label, randomized feasibility trial of home-based TEAS in individuals with IBD experiencing depressive symptoms. Based on the recommendations for feasibility and pilot studies [20], our target was to recruit 30 participants. The King’s College London Research Ethics Review Board approved this study (KCL Ethics Refs. HR/DP-23/24-37915). All the participants provided written informed consent.

Participants were recruited through online advertisements facilitated by the charity Crohn’s and Colitis UK (CCUK). The eligibility criteria included being 18 years or older; having a clinical diagnosis of CD, ulcerative colitis (UC), or another form of IBD; and experiencing at least mild-to-moderate depressive symptoms, defined as a score of 8 or higher on the Patient Health Questionnaire-9 (PHQ-9) (scale 0–27). This threshold was chosen based on meta-analytic evidence indicating that PHQ-9 cutoffs between 8 and 11 provide diagnostic accuracy for major depression in medical populations [21], and aligns with our aim to assess feasibility and acceptability in a broad at-risk group. Exclusion criteria were suicide risk (scoring 2 or 3 on the PHQ-9 question 9), active alcohol or drug dependency, the presence of a heart pacemaker, pregnancy or breastfeeding, and lack of access to smartphones or apps. Participants were not excluded if they had concomitant mental health conditions and did not receive financial incentives; however, the equipment, app, and training were provided free of charge.

The random allocation sequence was generated by CH using an online randomization service (simple randomization service [22]) employing block randomization to ensure balanced group sizes. Participants were randomly assigned to one of two groups: Group A, which received the TEAS intervention first, or Group B, which began the intervention 2 weeks after Group A’s treatment period was concluded. Allocation was concealed before randomization to prevent potential bias in group assignments. CH was responsible for enrolling participants and assigning them to the intervention groups.

This staggered implementation adhered to ethical principles by ensuring that both groups received treatment, while allowing for a comparison between immediate and delayed interventions. Participants continued their existing IBD and depression management during the TEAS intervention, including ongoing antidepressant medications, psychotherapy, or cognitive behavioral therapy. We requested that participants keep any depression therapy stable throughout the study.

Owing to the nature of the intervention, blinding was not feasible after group allocation.

### Feasibility outcomes

Feasibility outcomes included recruitment and retention rates, completion of outcome measures, and fidelity to the intervention protocol (see below).

### Safety, tolerability, and acceptability

Safety and tolerability were assessed by monitoring adverse events after the completion of the 21-day intervention sessions. The TEAS Adverse Events Questionnaire was developed on the basis of a comprehensive literature review [12,15].

An intervention acceptability questionnaire, adapted from Sekhon’s *et al.* [23] theory-informed framework, assessed seven constructs: affective attitude; burden; ethicality; perceived effectiveness; self-efficacy; opportunity costs; and general acceptability. Responses were measured using a 5-point Likert scale anchored from ‘completely acceptable’ to ‘completely unacceptable’.

The acceptability questionnaire was administered at two time points: baseline for both groups and postintervention (week 4 for Group A, week 9 for Group B). The postintervention version retained the same items as the baseline questionnaire, with the questions rephrased in the past tense. Additionally, the ‘affective attitude’ question was also modified to: ‘Would you recommend the TEAS intervention to others?’ Table 3 (in the Results section below) outlines the questionnaire items, scoring systems, and participant responses.

### Clinical outcomes

Online self-assessments were conducted at baseline and at weeks 4 and 9, following the initiation of the TEAS sessions (Table 1). The following validated rating scales were employed:

**Table 1.** Assessments and timing

Measure	Screening	Baseline (T0)	Week 4 (T1) (End of intervention for group A)		Week 9 (T2) (End of intervention for group B)	
			Group A	Group B	Group A	Group B
Demographics	✓					
PHQ-9	✓	✓	✓	✓	✓	✓
SF-12		✓	✓	✓	✓	✓
IBD-F		✓	✓	✓	✓	✓
BPI-SF		✓	✓	✓	✓	✓
AQ-B		✓				
AQ-AI			✓			✓
AEQ			✓			✓

AEQ, adverse effects questionnaire; AQ-AI, acceptability questionnaire after intervention; AQ-B, acceptability questionnaire baseline; BPI-SF, brief pain inventory – short form; IBD-F, inflammatory bowel disease fatigue scale; PHQ-9, patient health questionnaire; SF-12, 12-item short form health survey.

1. PHQ-9: A 9-item tool for assessing the presence and severity of depressive symptoms.
2. The 12-Item Short Form Health Survey (SF-12) [24] is a widely used measure of health-related quality of life. This condensed version of the SF-36 evaluates two primary domains: the physical component summary (PCS) and mental component summary (MCS).
3. The brief pain inventory – short form (BPI-SF) is [25] used for pain assessment and includes the following components: body diagram (for pain location), pain severity, and pain interference.
4. Inflammatory Bowel Disease Fatigue Questionnaire (IBD-F) [26]: the first section was used to evaluate the presence and severity of fatigue.

An open-ended questionnaire was also designed to gather participant feedback: Group A completed the questionnaire at weeks 4 and 9, whereas Group B completed it only at week 9. This approach aimed to identify any unexpected positive or negative effects of the treatment and to uncover unmet participant needs. All the data were collected remotely using a secure online REDCap platform.

### The intervention

**Fig. 1.**
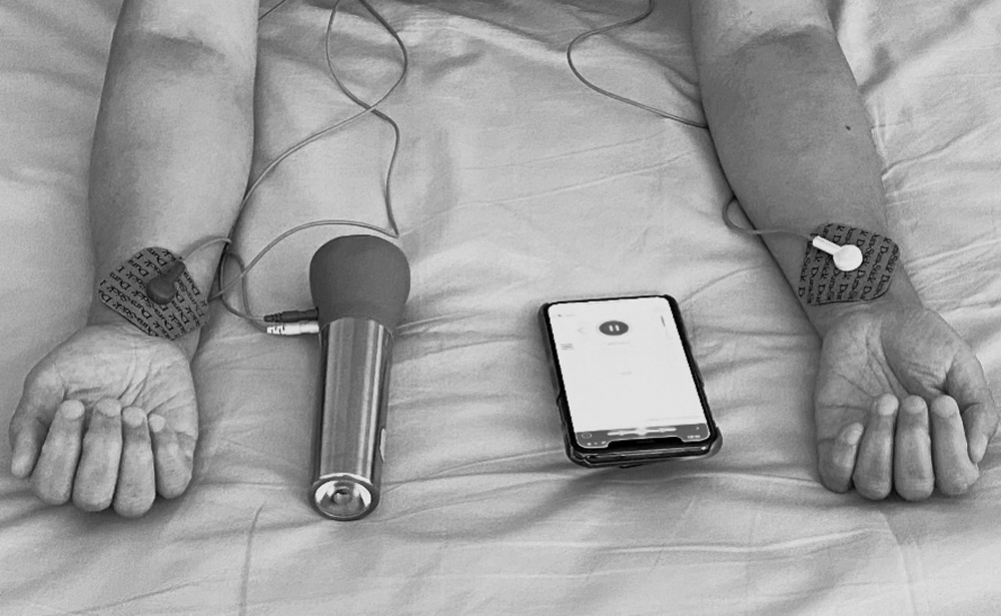
The TEAS device. TEAS, transcutaneous electrical acupoint stimulation.

A Raymedy (Netherlands) flashlight-sized stimulator (Fig. 1) was used to administer TEAS treatment once daily. The CE-certified device is operated using a mobile device. Each participant received the device, accompanying accessories, a user guide for the device and app, and Epaderm cream by mail, along with a prepaid return envelope to return the device.

Upon receiving the device, participants were offered one of four online training sessions via Microsoft Teams and an in-person option to accommodate their schedules. Most participants attended a small-group training session, and three participants received individual training because of scheduling conflicts. The sessions provided a brief overview of the research background and focused on teaching the participants how to use the device and app effectively. A training period of 3–4 days was allowed to practice using the TEAS system, after which most participants began the intervention period. In addition, we provided three short instructional videos demonstrating how to locate the acupuncture points.

**Fig. 2.**
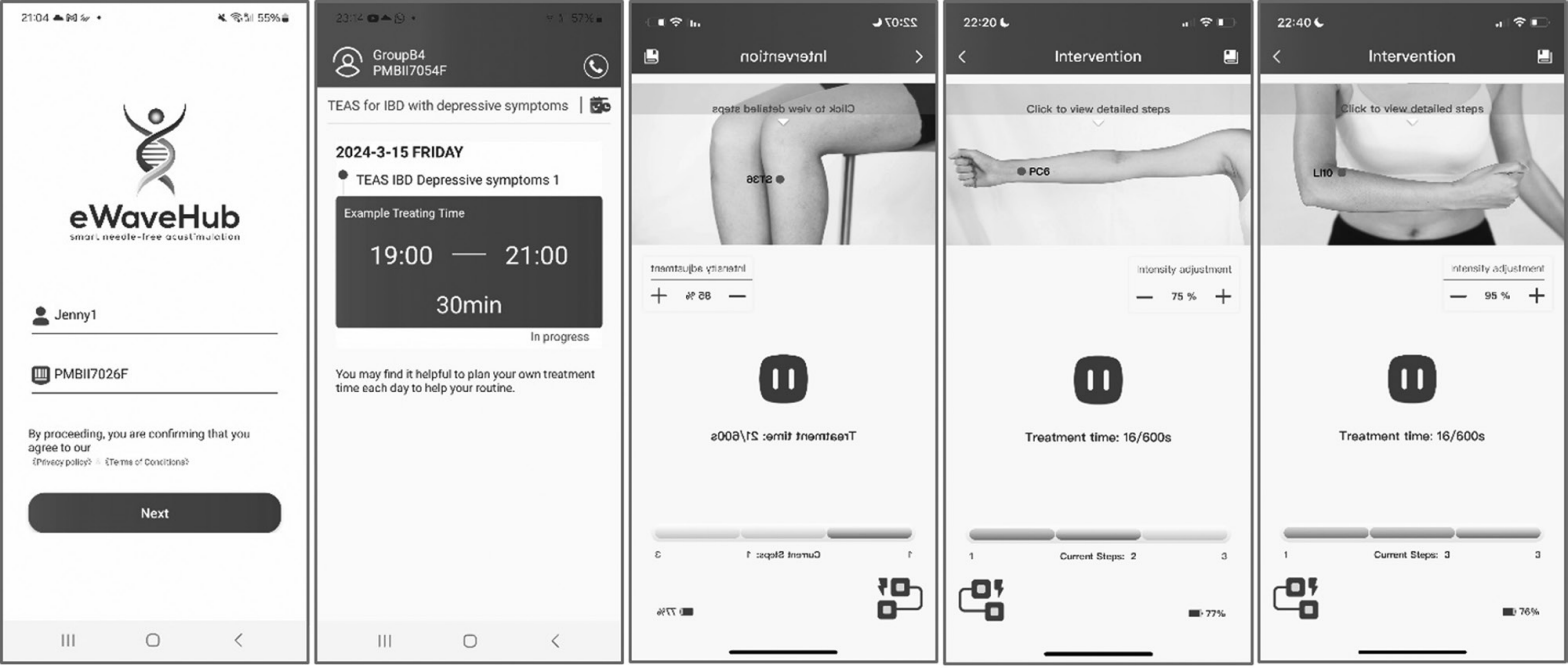
The app screenshots.

The device outputs and treatment processes were managed through the eWaveHub app, which is compatible with both iOS and Android smartphones. The app features images of acupoints, step-by-step treatment instructions, and automatic treatment duration, and allows users to adjust the output current intensity, ranging from 0 to 1 mA. The protocol targeted three pairs of acupoints: ST36 (ZuSanLi), LI10 (ShouSanLi), and PC6 (NeiGuan) (Fig. 2). Each pair of acupoints received 10 min of the specific pulse signal, with each daily session lasting for 30 min stimulation and up to 40 min in total, depending on the time required to switch between acupoints.

Participants followed the app’s guidance to position adhesive electrode pads on each pair of acupoints without the need to differentiate between positive and negative charges. Nine pulse waveform parameters were used to stimulate the acupoints, with three specific waveforms applied to each acupoint. The parameters were automatically updated every 7 days via the content management system, which also tracked intervention usage. Adherence to the intervention protocol was defined as completing the treatment on at least 18 of 21 days.

The app interface (Fig. 2) and functionality remained consistent throughout the intervention period. Key differences among the pulse waveform parameters include variations in the center voltage level, waveform complexity, and specific characteristics, such as the pulse width, amplitude, and frequency.

Participants could complete daily treatment at the time of their choice; however, we recommended administering TEAS between 19:00 and 21:00, approximately 1 h after dinner, as this timing is optimal for bowel disease interventions [17]. The daily treatment regimen automatically resets at midnight, with each dose logged and transmitted to the data management system. The app features daily reminders and technical assistance contact information. Additionally, participants received weekly WhatsApp messages encouraging communication and addressing any questions or concerns during the intervention period.

### Statistical analyses

No formal sample size calculation was performed for this feasibility study. Descriptive analyses were conducted using IBM SPSS Statistics (version 29.0) to examine the demographic characteristics and feasibility outcomes. Given that this feasibility study was not powered to detect differences between groups, no significance testing was undertaken.

The results are reported using the CONSORT framework for feasibility trials (Supplementary Data 1, Supplemental digital content 1, https://links.lww.com/EJGH/B190).

## Results

### Recruitment

**Fig. 3.**
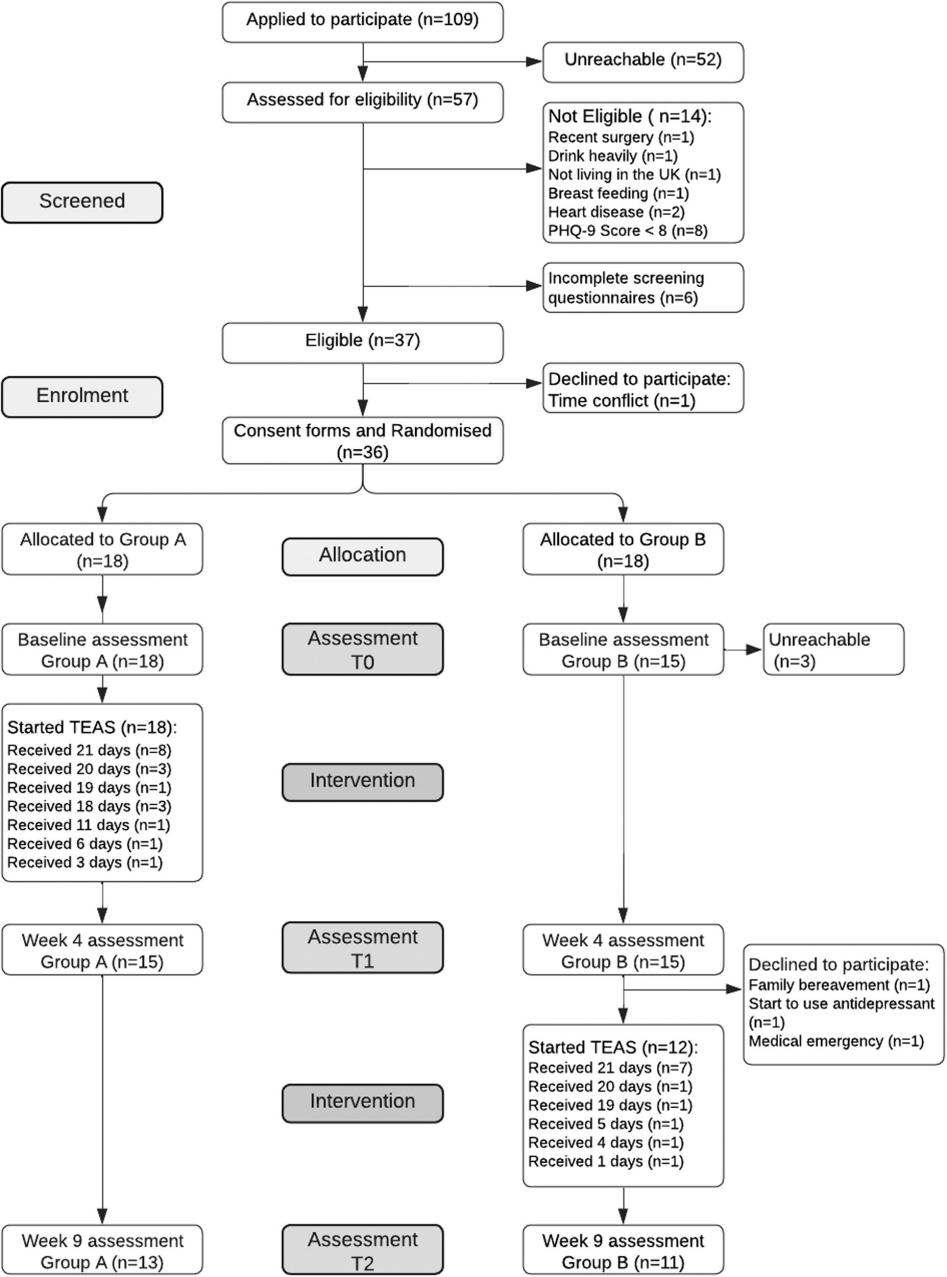
Consort diagram.

The flow of participants throughout the study is illustrated in the CONSORT diagram (Fig. 3). Following the announcement of the study recruitment on CCUK social media, 109 individuals with IBD expressed interest within 24 h and were provided with the Participant Information Sheet. Among the 57 individuals who completed the screening questionnaire, 37 (73%) met the eligibility criteria, and 36 of these eligible participants provided informed consent. One individual declined to participate, citing time-commitment concerns.

Eighteen participants were allocated to Groups A and B; however, three participants from Group B were unreachable for the baseline assessment. All 18 participants in Group A began the TEAS intervention. In both groups, 15 participants completed the Week 4 assessment with a combined completion rate of 83% (*n* = 30). In Group B, 12 participants started the TEAS intervention. By Week 9, 13 participants in Group A and 11 participants in Group B had completed the final assessment, resulting in a total of 24 participants and an overall retention rate of 67%.

### Baseline characteristics

The characteristics of the participants are summarized in Table 2. Of the 36 participants, 28 (78%) were women, and the mean age was 35.61 years (SD ± 10.32). The mean PHQ-9 depression score was 14.44 (SD ± 4.22), indicating moderate depression severity. Fourteen participants (39%) were taking antidepressant medication, and seven (19%) were undergoing counseling or CBT at the time of enrollment. No major differences in demographic or clinical characteristics, such as age, sex distribution, or depression scores, were observed between Groups A and B.

**Table 2.** Demographic and clinical characteristics of participants

Variable, *N* (%) unless otherwise specified	Group A	Group B
All	18	18
Age (years), mean (SD)	34.2 (10.39)	37.0 (10.35)
Sex		
Male, *n* (%)	5 (28)	3 (17)
Female, *n* (%)	13 (72)	15 (83)
Education		
Vocational/school level qualifications, *n* (%)	0 (0)	5 (27.8)
Advanced school level qualifications, *n* (%)	6 (33)	3 (16.7)
University degree (e.g. BSc, BA), *n* (%)	8 (44)	7 (38.9)
Postgraduate degree (e.g. MSc, PhD), *n* (%)	4 (22)	3 (16.7)
Relationship status		
Single, *n* (%)	11 (61.1)	7 (38.9)
In a relationship, *n* (%)	7 (38.9)	11 (61.1)
Smoking status		
Yes	0	1 (5.6)
Exsmoker, *n* (%)	5 (27.8)	7 (38.9)
No, *n* (%)	13 (72.2)	10 (55.6)
Type of IBD		
Crohn’s disease, *n* (%)	12 (66.7)	13 (72.2)
Ulcerative colitis, *n* (%)	6 (33.3)	4 (22.2)
Inflammatory bowel disease unclassified, *n* (%)	0	1 (5.6)
Months since IBD diagnosis, median (range)	58 (2–300)	74.5 (10–388)
Previous IBD surgery (yes), *n* (%)	5 (27.8)	4 (22.2)
On IBD biologic medications		
Yes, *n* (%)	12 (66.7)	14 (77.8)
No, *n* (%)	6 (33.3)	4 (22.2)
Months since depression symptoms, median (range)	50.5 (6–60)	48 (6–60)
Current treatment for depression		
Antidepressants, *n* (%)	8 (44.4)	6 (33.3)
Counseling/psychotherapy, *n* (%)	3 (16.7)	4 (22.2)
PHQ-9 Depression score, mean (SD)	13.9 (3.14)	15.0 (5.11)

IBD, inflammatory bowel disease.

### Feasibility outcomes

At baseline (T0), 33 participants (92%) completed the questionnaires, although one participant did not complete one of them. At week 4 (T1), 30 participants (83%) completed the assessment, and by week 9 (T2), 24 participants (67%) completed the questionnaires. However, some submitted online questionnaires were incomplete. For example, only 23 participants completed the adverse effects questionnaire.

### Intervention adherence

Adherence was tracked through the app’s management system and defined as completing at least 85% of the intervention sessions (18 of 21 days).

In Group A, 18 randomized participants (100%) initiated the intervention. Of these, 11 participants (61%) completed 20 or 21 sessions, while four participants (22%) completed 18 or 19 sessions, resulting in 15 participants (83%) meeting the adherence threshold. Three participants discontinued the intervention: one withdrew after the third session due to skin sensitivity, inability to tolerate even reduced stimulation intensity at 5%, another discontinued after six sessions due to a cold-like illness (uncertain if directly related to the intervention), and the third completed 11 sessions before being hospitalized for IBD. The mean adherence for Group A was 17.83 days out of 21.

In Group B, 18 participants were randomized, but only 12 (67%) began the intervention. Among those who started, 9 participants (50% of those randomized) completed 18 or more sessions, meeting the adherence threshold. Three participants discontinued the intervention: one because of a family illness, another because of an IBD symptom flare-up, and the third became unreachable. The mean adherence for Group B was 16.33 days out of 21.

**Fig. 4.**
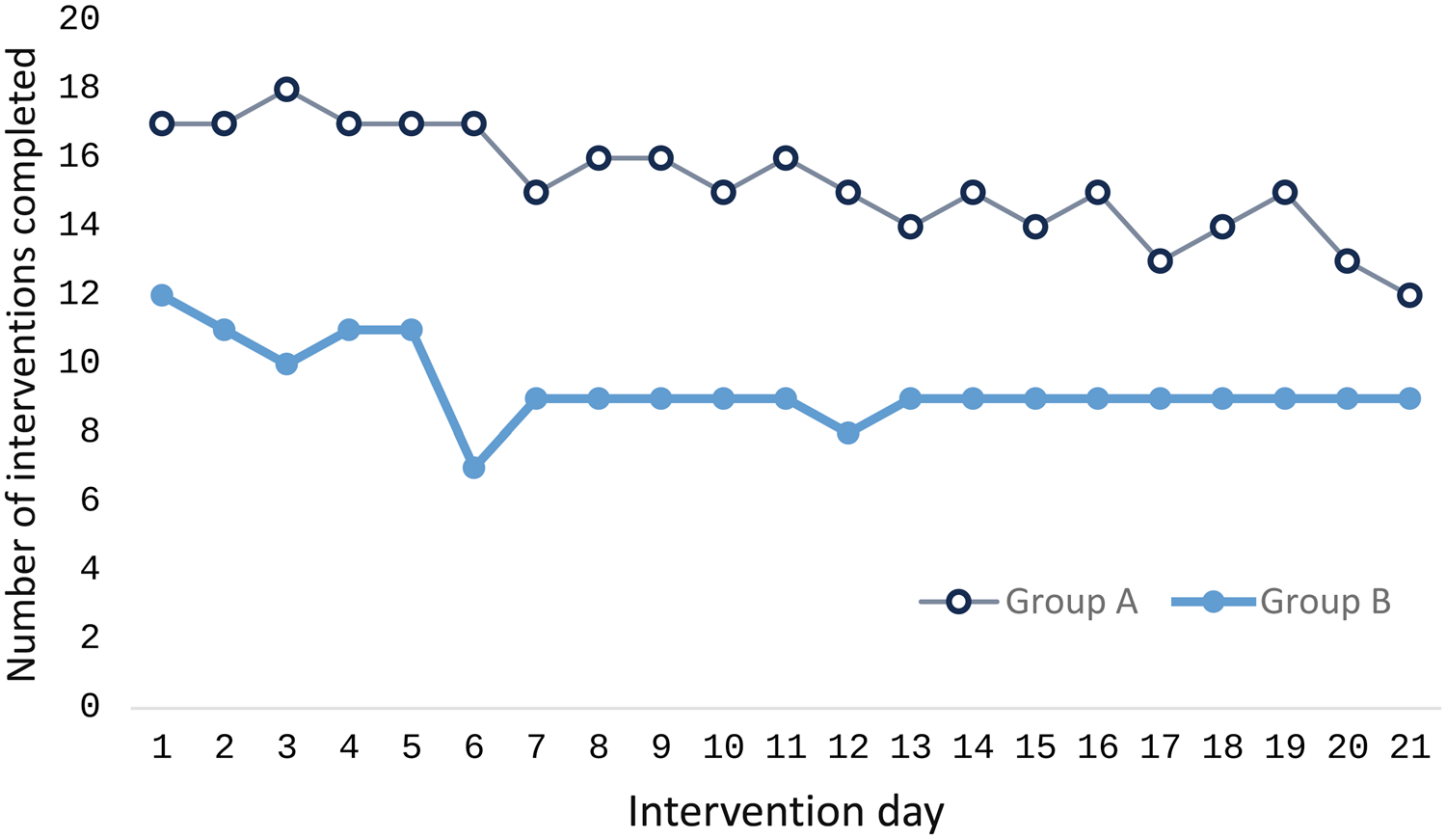
Daily TEAS intervention completion by group over 21 Days. TEAS, transcutaneous electrical acupoint stimulation.

Figure 4 illustrates adherence patterns over the 21-day study period. Group A demonstrated a gradual decline in adherence, while Group B showed a more consistent pattern following an initial adjustment period. Although both groups exhibited an overall reduction in adherence compared to initial levels, the patterns suggest potential differences in engagement trajectories that warrant further investigation in a larger sample.

### Safety, tolerability, and acceptability

Twenty-three participants completed the adverse effects questionnaire; the outcomes are summarized in Supplementary Data 2, Supplemental digital content 2, https://links.lww.com/EJGH/B191. One participant discontinued the TEAS intervention because of skin sensitivity. The most commonly reported side effects are skin redness, tingling, itching, and mild irritation at electrode sites. These effects were localized to the inner wrists and elbows, with no occurrence in the legs. They were generally mild to moderate in intensity and resolved shortly after the treatment session or the following morning.

Adjusting the current intensity reduced most skin reactions, and the cream (Epaderm) alleviated itching in most participants. Two participants reported that their skin issues resolved within 3 days after the intervention. Among those who experienced itching, 48% (*n* = 11) rated itching as mild, 13% (*n* = 3) as moderate, and 9% (*n* = 2) as severe.

One question asked, ‘Did you experience fatigue after daily intervention?’ Fifteen participants reported varying levels of exhaustion: seven rated their fatigue as mild, six as moderate, and two as severe. Multiple participants noted feeling drowsy during the 30-min sessions and indicated a need for rest following the intervention.

The acceptability ratings (Table 3) remained consistently positive across both time points, with no statistically significant changes (baseline median = 4, IQR = 1; postintervention median = 4, IQR = 1). Participants generally reported feeling ‘comfortable’ or ‘very comfortable’ with the treatment at both baseline and postintervention.

**Table 3.** Acceptability questionnaire and responses at baseline (*n* = 32), at the end of intervention (*n* = 26)

Question	Median (IQR)	Ratings *N* (%)				
		1	2	3	4	5
How do you feel about the TEAS treatment that uses a low electric current to stimulate acupuncture points?		Very uncomfortable	Uncomfortable	Neutral (no opinion)	Comfortable	Very comfortable
Baseline	4 (1)	0 (0%)	0 (0%)	8 (25%)	16 (50%)	8 (25%)
How much effort do you think you will need to put in for the TEAS intervention?/How much effort did you put into the TEAS treatments?		No effort at all	Neutral (no opinion)	A little effort	A lot of effort	Huge effort
Baseline	3 (0)	1 (3.1%)	4 (12.5%)	19 (59.4%)	7 (21.9%)	1 (3.1%)
Postintervention	4 (1)	1 (3.8%)	2 (7.7%)	5 (19.2%)	14 (53.8%)	4 (15.4%)
The TEAS intervention will improve my depression symptoms/The TEAS intervention has improved my depression symptoms		Strongly disagree	Disagree	Neutral (no opinion)	Agree	Strongly agree
Baseline	3 (1)	0 (0%)	0 (0%)	17 (53.1%)	15 (46.9%)	0 (0%)
Postintervention	4 (1)	0 (0%)	1 (3.8%)	7 (26.9%)	16 (61.5%)	2 (7.7%)
The TEAS intervention will improve my quality of life/The TEAS intervention has improved my quality of life		Strongly disagree	Disagree	Neutral (no opinion)	Agree	Strongly agree
Baseline	3 (1)	0 (0%)	0 (0%)	17 (53.1%)	14 (43.8%)	1 (3.1%)
Postintervention	4 (1)	0 (0%)	2 (7.7%)	7 (26.9%)	16 (61.5%)	1 (3.8%)
How confident do you feel about engaging with the TEAS intervention/How confident did you feel about using the TEAS intervention		Very unconfident	Unconfident	Neutral (no opinion)	Confident	Very confident
Baseline	4 (0)	0 (0%)	3 (9.4%)	0 (0%)	22 (68.8%)	7 (21.9%)
Postintervention	4 (1)	0 (0%)	2 (7.7%)	3 (11.5%)	11 (42.3%)	10 (38.5%)
The TEAS intervention will interfere with my other priorities/The TEAS intervention interfered with my other priorities		Strongly disagree	Disagree	Neutral (no opinion)	Agree	Strongly agree
Baseline	2 (0)	6 (18.8%)	18 (56.2%)	8 (25%)	0 (0%)	0 (0%)
Postintervention	2 (1)	3 (11.5%)	13 (50%)	4 (15.4%)	6 (23.1%)	0 (0%)
How acceptable is the TEAS intervention to you?/How acceptable was the TEAS intervention to you?		Completely unacceptable	Unacceptable	Neutral (no opinion)	Acceptable	Completely acceptable
Baseline	4 (1)	0 (0%)	0 (0%)	0 (0%)	19 (59.4%)	13 (40.6%)
Postintervention	4 (1)	0 (0%)	2 (7.7%)	1 (3.8%)	13 (50%)	10 (38.5%)
Would you recommend the TEAS intervention to others?		Strongly Not recommend	Not recommend	Neutral (no opinion)	Recommend	Strongly Recommend
Postintervention	4(0)	1 (3.8%)	1 (3.8%)	3 (11.5%)	15 (57.7%)	6 (23.1%)

Italics represent posttreatment phrasing.

The perceived effort required for the intervention increased slightly, with the median score rising from 3 (‘neutral’) at baseline to 4 (‘a little effort’) postintervention. This suggests that participants initially anticipated less effort or were unsure about the effort involved, but later found that the intervention required somewhat more commitment.

Despite this, the intervention was not seen as a major disruption to participants’ priorities. Postintervention, 62% (*n* = 16) of the participants disagreed or strongly disagreed that it interfered with other priorities. Additionally, the majority of participants expressed willingness to recommend the intervention to others, with 58% (*n* = 15) selecting ‘recommend’ and 23% (*n* = 6) selecting ‘strongly recommend’.

### Initial estimates of clinical outcomes

Table 4 presents descriptive statistics for key clinical outcomes, including means (± standard deviations) at baseline (T0), week 4 (T1), and week 9 (T2) for Groups A and B. Due to the small sample size, results are presented as observed trends, with emphasis on descriptive findings and clinical relevance rather than statistical significance.

**Table 4.** Clinical outcome measures at T0, T1, and T2

Outcome	Group	T0 (baseline)			T1 (week 4)			T2 (week 9)		
		Mean	SD	*N*	Mean	SD	*N*	Mean	SD	*N*
PHQ-9	Group A	13.94	3.35	18	7.73	3.69	15	9.54	3.95	13
	Group B	12.71	4.18	14	14.20	4.44	15	6.45	3.67	11
SF-12 PCS	Group A	38.26	9.69	18	39.75	12.73	15	38.16	8.30	13
	Group B	40.91	8.67	14	37.91	7.77	15	41.75	13.20	11
SF-12 MCS	Group A	32.01	4.87	18	43.26	6.63	15	41.78	9.79	13
	Group B	31.19	5.11	14	35.21	6.29	15	45.24	7.42	11
IBD-F	Group A	14.39	2.43	18	10.60	4.07	15	13.62	3.91	13
	Group B	14.14	2.93	14	14.73	3.10	15	12.45	4.01	11
BPI BodyDiagramTotal	Group A	3.83	2.04	18	2.80	2.04	15	2.85	1.99	13
	Group B	2.71	1.38	14	3.33	2.16	15	3.18	3.31	11
BPI PainSeverity	Group A	3.88	1.57	18	3.20	1.87	15	3.81	1.85	13
	Group B	4.02	2.07	14	3.63	2.07	15	2.86	1.82	11
BPI PainInterference	Group A	4.94	2.04	18	4.57	2.47	15	5.05	1.97	13
	Group B	4.67	2.54	14	4.87	2.80	15	3.34	2.78	11

BPI-SF, brief pain inventory – short form; IBD-F, inflammatory bowel disease fatigue scale; PHQ-9, patient health questionnaire; SF-12, 12-item short form health survey.

The severity of depression (PHQ-9) showed distinct temporal patterns between groups. Group A demonstrated a notable decrease from baseline (13.94 ± 3.35) to week 4 (7.73 ± 3.69), reflecting a 45% reduction. This improvement was partially attenuated at week 9 (9.54 ± 3.95), although scores remained below baseline. Group B showed a slight increase during the control period from baseline (12.71 ± 4.18) to week 4 (14.20 ± 4.44), followed by a marked improvement at week 9 (6.45 ± 3.67), indicating a substantial reduction in depression severity postintervention.

Health-related QoL (SF-12) provides summary scores for PCS health and MCS health. The physical health scores remained relatively stable across the time points in both groups. Group A showed a slight increase in PCS from baseline (38.26 ± 9.69) to week 4 (39.75 ± 12.73), followed by a return to near-baseline levels at week 9 (38.16 ± 8.30). Group B exhibited a minor decline during the control period from baseline (40.91 ± 8.67) to week 4 (37.91 ± 7.77), followed by improvement postintervention at week 9 (41.75 ± 13.20). Mental health scores demonstrated more substantial changes. Group A showed a marked increase in MCS from baseline (32.01 ± 4.87) to week 4 (43.26 ± 6.63), with improvements maintained at week 9 (41.78 ± 9.79). Group B demonstrated moderate improvement during the control period (baseline: 31.19 ± 5.11; week 4: 35.21 ± 6.29), followed by a substantial increase postintervention (week 9: 45.24 ± 7.42).

Fatigue severity (IBD-F) in Group A decreased from baseline (14.39 ± 2.43) to week 4 (10.60 ± 4.07), though scores partially rebounded at week 9 (13.62 ± 3.91) while remaining below baseline. Group B maintained stable scores during the control period (baseline: 14.14 ± 2.93; week 4: 14.73 ± 3.10), followed by improvement postintervention (week 9: 12.45 ± 4.01).

Pain measures (BPI-SF) revealed distinct patterns across dimensions. The total body diagram scores in Group A improved during the intervention (baseline: 3.83; week 4: 2.80) and remained stable (week 9: 2.85), whereas Group B showed improvement only postintervention. Pain severity scores in Group A remained stable (baseline: 3.88 ± 1.57; week 4: 3.20 ± 1.87; week 9: 3.81 ± 1.85), while Group B demonstrated a modest reduction postintervention (baseline: 4.02 ± 2.07; week 9: 2.86 ± 1.82). Pain interference scores in Group A remained consistent across all time points (baseline: 4.94 ± 2.04; week 4: 4.57 ± 2.47; week 9: 5.05 ± 1.97), while Group B showed stability during the control period (baseline: 4.67 ± 2.54; week 4: 4.87 ± 2.80) followed by improvement postintervention (week 9: 3.34 ± 2.78).

### Qualitative outcomes

In the open-ended questionnaire, 14 of the 26 respondents reported improved sleep duration and feeling more energetic upon waking. Two participants in Group A noted significant improvement following the intervention (week 4); however, due to heightened IBD disease activity (flare), they experienced a slight return of depressive symptoms by week 9. Users appreciated the device’s noninvasive nature and ease of use, despite some participants finding the daily 30 min of intervention challenging. Some participants reported connectivity issues with the app when operating other phone apps during treatment sessions. Overall, participants expressed appreciation that the intervention largely met their initial positive expectations. Most participants reported increased energy, longer sleep duration, enhanced alertness, improved focus, and reduced depressive symptoms (Supplementary Data 3, Supplemental digital content 3, https://links.lww.com/EJGH/B192).

## Discussion

The findings of this study provide insights into the feasibility and acceptability of implementing real-world interventions to improve mental health symptoms in IBD patients. The retention rate of 67% (24/36) supports the viability of large-scale trials.

The intervention demonstrated promising trends in improving mental health outcomes, with evidence of sustained effects in Group A and marked postintervention improvements in Group B. Following the intervention, both groups showed meaningful reductions in fatigue levels, particularly in Group B. Pain-related outcomes exhibited variable responses: Group B demonstrated improvements in pain interference postintervention, whereas Group A’s scores remained stable. The physical health outcomes remained relatively unchanged, suggesting that the intervention had a targeted impact on mental and emotional well-being.

Treatment response patterns differed between the groups. Group A exhibited strong initial improvements in mental health and depressive symptoms, although some regression occurred after the intervention ceased. Group B showed notable gains after intervention initiation at week 6, particularly in terms of mental health and fatigue measures. These preliminary findings suggest clinically meaningful improvements in depressive symptoms, fatigue, and QoL. However, the small sample size limits the generalisability of these results, indicating the need for larger studies to validate these effects.

The findings of this feasibility study highlight the potential of TEAS as an effective intervention to enhance mental health outcomes in patients with IBD. TEAS may serve as a viable treatment option for individuals with depression who have shown limited responses to conventional pharmacological or psychological therapies, face barriers in accessing traditional care, or seek complementary treatment approaches.

The study successfully met its recruitment targets within the anticipated timeframe, indicating both accessibility and willingness of the target population to participate. The considerable demand for depressive symptom interventions among individuals with IBD was demonstrated by the receipt of 109 expressions of interest within 24 h of posting social media advertisements. This robust response may be correlated with the high utilization of complementary medicine among patients with IBD, which previous studies have documented at rates ranging from 21 to 60% [27]. Furthermore, strong interest likely reflects the study’s emphasis on eliminating barriers to participation. Patients with IBD frequently face logistical challenges, including inflexible schedules and travel difficulties due to symptoms such as diarrhea, fatigue, and unexpected disease flare-ups [28]. The study’s fully remote design enhanced participant accessibility by addressing the common barriers to participation.

Participant feedback highlighted the acceptability of the TEAS intervention in terms of its usability and tolerability. Although the adverse effects were generally mild and transient, one participant discontinued the study because of a skin reaction. The high adherence rates to the intervention protocol demonstrated that the participants successfully integrated TEAS into their daily routines without a substantial burden. However, qualitative feedback indicated that some participants encountered challenges with daily adherence, particularly during IBD flares or due to time constraints. Technical issues with the mobile app emerged as a potential barrier, especially when participants attempted to use other apps during treatment sessions. Notably, the intervention’s noninvasive nature and flexibility of home-based administration served as key facilitators, supporting sustained participation and engagement.

Younger individuals may be more receptive to app-based interventions than older individuals. The prevalence of adolescent IBD is gradually increasing, with a 94% increase in incidence [1]. Patients with IBD often have ongoing medical and psychological needs [29], and home-based interventions provide a convenient way for them to access interventions. Digital acupuncture therapeutics present new opportunities to deliver care in an accessible format, reaching individuals who are unresponsive to medication or psychological therapy or those actively seeking complementary healthcare support.

Qualitative feedback highlighted unexpected positive outcomes such as improved sleep quality, energy levels, and alertness. These findings underscore the potentially broader impact of the intervention beyond its primary target of reducing depressive symptoms. Sleep disturbances are closely associated with IBD activity [30] and may increase the risk of flares in patients with Crohn’s [31]. In our study, ≥50% of the participants reported perceived improvements in sleep quality, including reduced time to fall asleep, increased sleep duration, and feeling more energetic upon waking. A colitis mouse model study suggested that electroacupuncture at acupoint ST36 might improve sleep fragmentation by targeting intestinal tight junction proteins, which could be a key mechanism underlying its effects [32].

Stimulation parameters are critically important for TEAS, as their effects are centrally mediated; the brain processes and interprets signals from TEAS based on these specific parameters [33]. The parameters used in this study were carefully designed. Moreover, the effectiveness of bilateral and unilateral TEAS remains unclear [34]. In this study, bilateral TEAS was performed.

These results suggested lower depression and fatigue scores. According to the meridian theory, IBD is closely related to disorders of the stomach meridian and the large intestine meridian. ST36 is the most frequently used acupoint in clinical manual acupuncture studies on CD and UC [35]. Although LI10 is less commonly used in clinical studies, recent research in mice has revealed the pathways and mechanisms underlying the antiinflammatory properties of ST36 and LI10 [13]. Both ST36 and LI10 are located on the distribution of PROKR2Cre nerve fibers, and low-intensity electrostimulation at these points can activate the vagal-adrenal antiinflammatory axis. PC6 is the most common acupoint for psychiatric disorders [14]. Stimulation of PC6 may modulate activity in cortical and limbic brain regions [36] and trigger vasodilation in the middle cerebral artery [37]. TEAS at PC6 has been shown to reduce high-sensitivity C-reactive protein levels [11]. Therefore, stimulation of ST36, LI10, and PC6 may have specific efficacy in suppressing systemic inflammation and potentially creating synergistic effects to relieve depressive symptoms.

Despite its high prevalence, few interventions for managing fatigue in IBD have been described, and no therapies have demonstrated consistent efficacy [38]. However, both needle acupuncture and electroacupuncture are effective in managing fatigue in IBD patients [39]. Furthermore, IBD patients with fatigue exhibit distinct immune profiles characterized by a chronically active and Th1-skewed immune system, even during clinical remission [40]. This immune dysregulation may explain why our study also demonstrated improvements in fatigue severity.

### Limitations

As a feasibility study, this research was not designed or powered to detect clinical differences between groups. While the intervention demonstrated potential benefits in improving mental health and alleviating fatigue symptoms, the small sample size limited statistical power. This limitation is reflected in the response variability observed in the standard deviations and wide confidence intervals, highlighting the uncertainty in the precise magnitude of between-group differences. Given these constraints, the findings should be interpreted as preliminary descriptive trends rather than as definitive outcomes.

Although the results provide preliminary evidence of the feasibility and potential effectiveness of the intervention, several limitations of this study warrant consideration. During the SF-12 QualityMetric scoring process, question 4b (item RE3) was identified as having been modified, necessitating its exclusion from the dataset, following QualityMetric’s scoring guidelines. Although this omission did not affect the calculation of PCS or MCS scores, it represents a deviation from the standardized SF-12v2 protocol and may limit comparability with studies using the unmodified survey. Additionally, this study lacked professional clinical assessments of depression, measurements of IBD disease activity indices, and evaluation of biological markers. Given the growing evidence supporting the beneficial effects of acupuncture on intestinal inflammation [34], future research should incorporate immune activity markers such as fecal calprotectin [41] or C-reactive protein levels [42] to assess inflammatory activity. As a small, unfunded feasibility study, we were not able to fund these objective tests. Future studies would benefit from this, as well as the measurement of subjective self-reported disease activity scores. Larger-scale studies are also needed to validate these preliminary findings and more accurately estimate treatment effects. Longer follow-up should be used in future studies to check if any changes are sustained. A future larger or longer study should also monitor any changes in other therapies for depression and in IBD medication. We did not exclude people with mental health conditions other than depression: this could potentially confound the results. We acknowledge that the drop-out rate was high in group B before commencing the intervention, and do not have data on the reasons for this.

The intervention relies on users correctly placing adhesive electrode pads on acupoints. Despite app-based guidance, user errors in locating and adhering to pads can compromise treatment effectiveness and consistency. Moreover, the TEAS intervention involved multiple variable parameters, and the 21-day intervention period may have been too short to fully evaluate the long-term effects. A shorter duration may limit therapeutic impact, particularly for chronic or complex conditions.

Participants had varying tolerances to electrical stimulation, and the intervention allowed adjustment of current intensity (0–1 mA) based on individual comfort. While this approach introduced variability in stimulation dose, it aligns with standard practice in neuromodulation research, including taVNS studies in depression [43], where stimulation is typically set below the pain threshold but remains perceptible. Some participants with lower tolerance may have received a lower dose, and others may have approached the device’s upper limit. Although we did not formally assess the relationship between stimulation intensity and clinical outcomes in this feasibility study, future trials should consider stratified or standardized stimulation protocols to explore this further. Furthermore, pulse waveform parameters were standardized and automatically adjusted every 7 days, but individual patient responses or progress were not assessed at these intervals. The current TEAS literature has yet to establish a consensus on the optimal parameters for treating IBD patients with depression, highlighting the need for further investigation in this area.

## Conclusion

TEAS stimulation at PC6, LI10, and ST36 using the specified parameters appears to be feasible remotely. TEAS may serve as an effective adjunct to standard drug therapy in patients with IBD, offering a novel approach to self-management. The TEAS intervention and research protocol were feasible and well accepted by patients, with preliminary indications of clinical benefits. A full-scale randomized controlled trial is warranted to rigorously evaluate the effectiveness of TEAS in managing depression among patients with IBD, with the addition of measures to enhance engagement in a waitlist control group.

## Acknowledgements

The authors thank the Research and Quality Improvement Department of Crohn’s & Colitis, UK for supporting recruitment.

This work was supported by a free equipment loan and technical support from the MDB Therapy Company, utilizing Raymedy Company technology. Raymedy was not involved in the acquisition, analysis, or interpretation of the data.

All authors approved the final draft of the manuscript. All authors were involved in writing and revising the manuscript.

C.H.: conception of the work, data collection, analysis, and writing the original draft. C.N.: methodological assessment, supervised the entire study process, and provided critical revisions to the manuscript. W.C.D.: data analysis and provided critical revisions to the manuscript.

### Conflicts of interest

Huang initially founded MDB Therapy in collaboration with Raymedy to conduct the TEAS research project. Following the successful completion of the study, both parties agreed to grant MDB Therapy the right to further develop and commercialize Raymedy technology in the UK. C.N. speaker fees from Janssen, WebMD, Medscape, Merck Pharmaceutical, Tillotts Pharma UK, and Lilly. Pfizer advisory board. No funding was received personally or at the institution involved in this study. W.C.-D. speaker fees from Dr. Falk Pharma and research funding from Bristol Myers Squibb and Crohn’s and Colitis UK. No funding was received personally or at the institution involved in this study.

## Supplementary Material


