# Supplementary material for: Home-based transcutaneous electrical acupuncture-point stimulation for depressive symptoms in inflammatory bowel disease: a randomized feasibility study
Source: Eur J Gastroenterol Hepatol. 2025 Oct 29;37(12):1326–36. doi: 10.1097/MEG.0000000000003034 (PMC12577661; doi:10.1097/MEG.0000000000003034)
Supplement: Supplementary file 2 [file ejgh-37-1326-s002.docx]

Supplemental Data 2

| **The Adverse Effects Questionnaire. The Adverse Effects n (%)**  **Total response n=23** | | | | |
| --- | --- | --- | --- | --- |
| Did you experience Tingling during the intervention? | | | | |
|  | Absent | Mild | Moderate | Severe |
|  | 5 (21.8) | 7 (30.4) | 9 (39.1) | 2 (8.7) |
| Is this related to TEAS? | |  |  |  |
|  | Definitely | Possible | No |  |
|  | 15 | 2 | 1 |  |
| Did you experience Burning? | |  |  |  |
|  | Absent | Mild | Moderate | Severe |
|  | 16 (69.6) | 6 (26.1) | 0 | 1 (4.3) |
| Is this related to TEAS? | |  |  |  |
|  | Definitely |  |  |  |
|  | 7 |  |  |  |
| Did you experience Skin Bruising? | | | | |
|  | Absent | Mild | Moderate | Severe |
|  | 20 (87) | 3 (13) | 0 | 0 |
| Is this related to TEAS? | |  |  |  |
|  | Definitely |  |  |  |
|  | 3 |  |  |  |
| Did you experience itching? | |  |  |  |
|  | Absent | Mild | Moderate | Severe |
|  | 7 (30.4) | 11 (47.8) | 3 (13) | 2 (8.7) |
| Is this related to TEAS? | |  |  |  |
|  | Definitely | Possible | No |  |
|  | 13 | 2 | 1 |  |
| Did you experience Blood blister? | | | | |
|  | Absent | Mild | Moderate | Severe |
|  | 21 (91.3) | 2 (8.7) | 0 | 0 |
| Did you experience pain? | |  |  |  |
|  | Absent | Mild | Moderate | Severe |
|  | 15 (65.2) | 3 (13) | 4 (17.4) | 1 (4.4) |
| Did you experience Sore muscles? | | | | |
|  | Absent | Mild | Moderate | Severe |
|  | 19 (82.6) | 3 (13) | 1 (4.4) | 0 |
| Did you experience Dizziness or Headaches? | | | | |
|  | Absent | Mild | Moderate | Severe |
|  | 15 (65.2) | 4 (17.4) | 2 (8.7) | 2 (8.7) |
| Is this related to TEAS? | |  |  |  |
|  | Definitely | Possible | Unlikely |  |
|  | 2 | 5 | 1 |  |
| Did you experience Heart palpitations or abnormal heart beats? | | | |  |
|  | Absent | Mild | Moderate | Severe |
|  | 21 (91.3) | 2 (8.7) | 0 | 0 |
| Did you experience Discomfort during the intervention? | | | | |
|  | Absent | Mild | Moderate | Severe |
|  | 14 (60.9) | 6 (26.1) | 1 (4.3) | 2 (8.7) |
| Is this related to TEAS? | |  |  |  |
|  | Definitely | Possible | No |  |
|  | 6 | 2 | 1 |  |
| Did you experience Fatigue after daily intervention? | | |  |  |
|  | Absent | Mild | Moderate | Severe |
|  | 8 (34.8) | 7 (30.4) | 6 (26.1) | 2 (8.7) |
| Is this related to TEAS? | |  |  |  |
|  | Definitely | Possible | Unlikely | No |
|  | 1 | 7 | 4 | 3 |
