# Supplementary material for: Home-based transcutaneous electrical acupuncture-point stimulation for depressive symptoms in inflammatory bowel disease: a randomized feasibility study
Source: Eur J Gastroenterol Hepatol. 2025 Oct 29;37(12):1326–36. doi: 10.1097/MEG.0000000000003034 (PMC12577661; doi:10.1097/MEG.0000000000003034)
Supplement: Supplementary file 3 [file ejgh-37-1326-s003.docx]

# Supplemental Data 3

**The post-intervention open-ended questionnaire**

**Total response n=26**

| **Q1** During the treatment, apart from the intervention's impact on your level of depression (if any) Sleep | | |
| --- | --- | --- |
| Detrimental impact | No noticeable impact | Beneficial impact |
| 1 (3.85) | 11 (42.31) | 14 (53.85) |
| **Q2** During the treatment, apart from the intervention's impact on your level of depression (if any) Energy level | | |
| Detrimental impact | No noticeable impact | Beneficial impact |
| 0 | 12 (46.15) | 14 (53.85) |
| **Q3** During the treatment, apart from the intervention's impact on your level of depression (if any) Tiredness | | |
| Detrimental impact | No noticeable impact | Beneficial impact |
| 0 | 16 (61.54) | 10 (38.46) |
| **Q4** During the treatment, apart from the intervention's impact on your level of depression (if any) Skin condition | | |
| Detrimental impact | No noticeable impact | Beneficial impact |
| 7 (26.92) | 19 (73.08) | 0 |
| **Q5** During the treatment, apart from the intervention's impact on your level of depression (if any) Ability to function | | |
| Detrimental impact | No noticeable impact | Beneficial impact |
| 0 | 12 (46.15) | 14 (53.85) |
| **Q6** During the treatment, apart from the intervention's impact on your level of depression (if any) Appetite and digestion | | |
| Detrimental impact | No noticeable impact | Beneficial impact |
| 2 (7.69) | 18 (69.23) | 6 (23.08) |

| **Q 7** | **Please tell us what aspects of the study were the most successful** | |
| --- | --- | --- |
|  | **Study ID** | **Participants quotes** |
|  | A3 | Felt more motivated and energised. Relaxing doing the treatment. |
|  | A5 | Massively increased energy in week two, but only around four hours’ sleep each night, which I think left me run down. Week three I slept for seven to eight hours a night and feel really refreshed. My mood is much improved, but it could be linked to the increased energy. I’m not sure. I feel my Crohn’s is slightly improved as well and also my arthralgia, especially in week two. |
|  | A8 | In terms of the actual effort needed to do the treatment I would say this is the most successful as it’s really easy and straightforward to do. |
|  | A12 | The treatment made me sleepy whilst using which helped me to get a better night’s sleep. |
|  | A17 | Very easy to understand and use the system, relaxing to take part. |
|  | A2 | There was a noticeable increase in the quality of my sleep as this study progressed. |
|  | A1 | Using the device was easy, simple and non-invasive. |
|  | A9 | I had more energy; I woke up feeling I had less pain and was more alert throughout the day. I felt less depressed and also that I had a better quality of sleep and slept deeper. I enjoyed taking some time out each day specifically for myself. |
|  | A4 | Unfortunately, I didn’t see a benefit in my fatigue which I was hoping for, although I did seem to get better quality sleep. |
|  | A14 | I felt my sleep was better and deeper. |
|  | A16 | I feel the treatment gave me more energy on a morning and I was less tired throughout the day. |
|  | A18 | More awake and alert. |
|  | A11 | I felt more energised when waking up. |
|  | A15 | First two weeks and particularly first two days where I was the most positive. I cannot remember being in years and had a lot more energy. |
|  | A18 | More intense sleep. |
|  | B9 | I didn't find any aspect successful. |
|  | B12 | Feeling sleepy and sleeping really well, uninterrupted and feeling less low in mood. |
|  | B13 | Depression reduced. |
|  | B6 | I don't think I experienced negative side effects and may have had benefits. I think my mood was generally positive throughout. I didn't encounter a flare of my Crohn's disease; however, my symptoms are currently in remission via my treatment already. |
|  | B1 | I definitely felt that my mood increased a lot. I haven’t felt anywhere near as depressed these last few weeks. |
|  | B4 | All aspects of the study were successful, I have felt an overall improvement of general wellbeing/mood as well as improved Crohn's symptoms too. |
|  | B2 | Possible improvement in mood but fluctuating. |
|  | B8 | It was fun to try out the TEAS device. |
|  | B7 | I have issues getting to sleep. I fell asleep very quickly and didn't wake as often. |
|  | B11 | I found I slept better each night after the treatment and post treatment to date. My appetite has stabilised, and I am no longer needing to eat as much as I did, so overall, digestion is better than it was. |
|  | B5 | I felt much better within myself which is very much appreciated. |
| **Q8** | **Please tell us about what aspects of the study were the least successful** | |
|  | **Study ID** | **Participants quotes** |
|  | A3 | During the last week of the treatment, I became unwell with my Crohn’s disease which led to vertigo and fainting. I’m not sure if the treatment affected this as I haven’t had those symptoms before. |
|  | A5 | I did get quite run down but it could be because the increased energy led me to do more than I usually would. |
|  | A8 | Personally, I couldn’t cope with the sensation of the treatment through the pads (prickly sharp sensation that eventually became painful rather than just discomfort) and even with the Epaderm. I was experiencing the itching beneath the surface that usually comes before psoriasis breakouts. Stopping the treatments meant the red itchy patches didn’t break out though. |
|  | A12 | Side effects from the pads - itchy skin; redness etc. unfortunately I can’t say I’ve noticed a huge difference to my depression symptoms, however other factors in my personal life may have prevented any change from being noticed. |
|  | A17 | Some days it was hard to ensure I could fit it into my schedule. |
|  | A23 | I have very busy days, which often means the only time I get to relax and wind down is very close to midnight, which actually made the use of the product slightly stressful, as it felt as if I had to complete something within a strict timeframe. |
|  | A1 | I didn’t struggle as didn’t have any particular plans, but it may be difficult for some people to do between 7-9pm daily (if this was a daily ongoing intervention) and drink so much water afterwards (just before sleeping). |
|  | A9 | I had mild skin conditions where the pads were placed, I found it hard to know if I had found the correct positioning for the pads and I found I couldn't turn the percentage up very high, sometimes only managing 30% because the treatment was painful. I had headaches on most days after the treatment and my stomach issues were all over the place during the three weeks whereas previously, they were fairly stable (although I did receive my Stelara injection during treatment so may be due to this). I had a lot of fatigue during treatment. |
|  | A4 | Treating mood and fatigue. |
|  | A14 | The skin irritation from the electrical current and the pads. The skin on one wrist and one arm was very sensitive and the treatment was slightly painful at times, despite turning the intensity down. There was a lot of irritation from the pads - itching, redness, sore, little bumps. The cream helped but it did not fully go until next morning. |
|  | A16 | I suppose the itchiness of the pads but applying the cream did help this. |
|  | A6 | Sleep, however I’ve never been a great sleeper. |
|  | A11 | My skin, which already sometimes had bad rashes, became dry and very itchy. |
|  | A15 | Unfortunately, it has sent me into a Crohn’s flare which started during the last week of treatment and is currently ongoing. |
|  | A18 | Occasional skin rash. |
|  | B9 | I found the treatment very difficult to tolerate even on the lowest setting of 5%. |
|  | B13 | The pads didn't stay sticky for very long, the app isn't the most easy to use as has to run in the foreground and you can't use your phone at the same time or it doesn't record your treatment. |
|  | B6 | A few times the equipment seemed to "hang up" and I couldn't get it to reset without taking me back to the first Acupuncture point under my knee when I'd already completed that. Then it didn't always record that I'd completed that day's session as a result. |
|  | B1 | There were no down sides for me, only the itching while using the device. |
|  | B4 | There were some small technical issues on occasion with the app mainly with connecting to Bluetooth, but not anything too difficult to quickly rectify. I do also need to flag that since the hour time change the app has logged my treatment times an hour earlier than when I actually did the treatment. |
|  | B2 | I tend to go to bed quite early (around 9pm), so even starting the treatment at 7pm, the water drunk after treatment often kept me up later than normal. |
|  | B8 | It is difficult to notice if TEAS improved level of depression, tiredness etc. |
|  | B7 | Pain during the usage, I seem to have very sensitive skin. I react to smart watches also. |
|  | B11 | My fatigue didn't seem to improve. |
| **Q9** | **Are there any suggestions for improving the study or the treatment?** | |
|  | **Study ID** | **Participants quotes** |
|  | A5 | I’d he really interested to know if the effects are due to last for a period after the treatment and whether ongoing treatment would be three weeks every six months or some other frequency - and whether traditional acupuncture on the same acupuncture points would have the same effect as this treatment? |
|  | A8 | Maybe allow the app to run in the background so patients could meditate or do emails etc. |
|  | A2 | Ensure I can use other apps on my phone whilst the programme is running. |
|  | A1 | Perhaps a longer study (6-8 weeks) - I’m happy to take part in another study if you run one, thanks! |
|  | A6 | Maybe checking that the pads were placed correctly. I got more confident in that with the support from the study researcher. |
|  | A11 | I can't think of any. |
|  | A15 | Longer for each different phase to differentiate treatments effects and whether coincidence plays a part. |
|  | A18 | More concise instruction on % strength to use. |
|  | B13 | Improve the app. |
|  | B6 | The smoothness of the transition between the 3 Acupuncture points so that the tool seamlessly continues through. |
|  | B1 | No, it was really good. |
|  | B4 | Smoother app. |
|  | B8 | A way to notice/measure symptoms throughout the study. |
|  | B7 | A better diagram of placement. Sometimes I wondered if I had it in the wrong area although I'm sure I had it right from the notes I took with the online meeting beforehand. |
|  | B11 | To have the treatment for a longer period. I think if I did have that I would see additional improvements and benefits. |
|  | B5 | I'm quite hairy so I shaved my acupoints which improved contact with my skin and therefore made the treatment more effective. |
| **Q10** | **Do you have any other comments or feedback regarding the study?** | |
|  | **Study ID** | **Participants quotes** |
|  | A5 | I’m very grateful to have had the opportunity to participate. |
|  | A8 | The study is a brilliant idea and although it’s not worked for me, I can absolutely see how it could be beneficial for others. Keep up the good work! |
|  | A17 | Just a big thank you to the study researcher. |
|  | A2 | Often didn't feel the electrical stimulation despite being on maximum power, perhaps providing an increased power to what is already available? |
|  | A1 | Brilliant study, thank you so much, you have been extremely helpful. I have enjoyed taking part.  Unfortunately, during the study, I was hospitalised due to ulcerative colitis for 2 days (I took the device with me and completed all treatments), but my pain was fluctuating due to my flare up, and then I started a new biologic medication (infliximab) during the study - this medication caused fatigue. Since my pain / symptoms / fatigue / medications were fluctuating during the study, it was difficult for me to truly draw any solid conclusions about the TEAs device (I feel more down with pain, but my pain was extremely variable during the study due to my flare and medication change). So, I can’t conclude properly if the device made a significant difference or not, since my health was extremely variable during the study. |
|  | A9 | Overall, my experience was very positive, and I would definitely use this treatment in future. If renting the device becomes a possibility I would continue with the treatment as I do believe it has made a positive difference. |
|  | A14 | No, study was well run, and good amount of contact with the study researcher. |
|  | A6 | Would definitely get a machine if they were available. |
|  | A11 | I am really glad I participated so thank you! |
|  | A15 | Hopeful that this can help people in the future. |
|  | A18 | Suggest using just before going to bed as it makes you feel tired. |
|  | B12 | Weirdly I enjoyed the tingling almost pins and needles feeling whilst in the 2nd week of the treatment. however during the 3rd, this feeling subsided and stopped and became a little stingy. |
|  | B13 | It was great. |
|  | B6 | I'm wondering if it would have been good to complete shirt Patient Reported Outcomes per weekly current level change. It would give an opportunity to breakdown the results more. |
|  | B1 | No, it was great! |
|  | B4 | I have really enjoyed taking part in the study and can say there are benefits in both Crohn’s symptoms and my mood/wellbeing. I look forward to seeing any results available from the study moving forward. |
|  | B8 | The study researcher was very supportive. |
|  | B11 | It was great and exceeded my expectations. Thank you. |
|  | B5 | Thank you! The treatment helped my depression which is amazing! Best of luck with the study. |
| **Q11** | **If you have any additional remarks or feedback after 4 -week you completed the TEAS study, please kindly share them below: (Only for Group A)** | |
|  | **Study**  **ID** | **Participants quotes** |
|  | A5 | Hard to assess direct or indirect benefits or effects in tome period. Doing the treatment daily could sometimes feel a little restrictive if I had a busy schedule, however it was still more convenient than booking or travelling to an Acupuncture clinic. |
|  | A8 | It really really helped with my mood. I think it was great! |
|  | A2 | I feel like the benefits of the trial have gone completely now and I’m back to how I was before. I’ve had a bad flare up too, so that probably hasn’t helped. |
|  | A1 | Thank you for the opportunity! |
|  | A6 | The first and second part were extremely beneficial but during the 3rd adjustment, I started going into a flare. |
|  | A11 | I think it had help and would continue to if I can use it regularly. |
|  | A15 | Thank you so much for everything, I really appreciate it!! :) |
|  | A18 | Thank you! I enjoyed the study and found it beneficial for my depression. The study was well organised, instructions were easy to follow, and the study researcher was very supportive. |
